# S231. THE ROLE OF DOPAMINERGIC AND GLUTAMATERGIC NEUROTRANSMISSION IN DELUSIONAL IDEATION AND SENSORY INFORMATION PROCESSING OF PATIENTS WITH SCHIZOPHRENIA IN COMPARISON TO HEALTHY HUMAN PARTICIPANTS

**DOI:** 10.1093/schbul/sby018.1018

**Published:** 2018-04-01

**Authors:** Wolfgang Strube, Graziella Quattrocchi, Simon Little, Louise Marshall, Alkomiet Hasan, Sven Bestmann

**Affiliations:** 1 University of Munich, LMU; 2 Sobell Department, UCL; 3 Wellcome Trust

## Abstract

**Background:**

The primary aim of this study was to generate neurobiological evidence regarding the impact of dopaminergic and glutamatergic neurotransmission on reasoning biases related to delusional ideation in patients with schizophrenia associated with impaired processing of sensory information. The proposed respective roles of these neurotransmitter systems have been encapsulated in the so-called dopamine- and glutamate-hypotheses of schizophrenia. From a behavioural perspective both reduced glutamate and enhanced dopamine levels are currently discussed as critical contributing factors to generate aberrant beliefs (glutamate) during information sampling and to generate confidence or expected precision (dopamine) during action selection. Hence, by modulating levels of glutamate and dopamine in the brain we hypothesized to induce reported impairments of patients with schizophrenia related todelusional ideation.

**Methods:**

The study consisted of three aligned experiments: In the first two experiments a prospective interventional drug study was conducted with n=192 participants employing a randomized, placebo-controlled, double-blinded design on two parallel testing-groups, receiving either dopaminergic or glutamatergic neuromodulators: Experiment I: either 2.5mg haloperidol (D1/D2-receptor antagonist; HAL), 2.5mg bromocriptine (D2-receptor agonist; BRO), or placebo (PLC-1). Experiment II: either 120mg Dextromethorphan (NMDA-receptor antagonist, DXM), 250mg D-Cycloserine (NMDA-receptor agonist, CYC), or placebo (PLC-2). In the third experiment n=45 patients with schizophrenia (SZ) and n=45 healthy control participants (HC) matched for gender, age and IQ were investigated. All experiments employed a computerized (Matlab, Cogent) version of the Beadstask (Huq, Garety et al. 1988). In total participants processes 60 Beadstask trials subdivided into three levels of difficulty: (I) easy trials with a bias of 80–90% for one predominant bead color in a sequence, (II) difficult trials (60–70% bias), and (III) ambiguous trials (no bias, 50% likelihood). Additionally, the task consisted of three parts that were presented in a fixed order: an easy draws-to-decision condition, an easy probability estimates condition, and a difficult draws-to-decision condition.

**Results:**

In accordance with foregoing studies, SZ patients showed significantly less draws to decision compared to HC (all p≤0.038). Explorative analysis across experimental conditions further revealed no significant differences for participants receiving DXM (NMDA-receptor antagonist) compared SZ patients (all p≥0.090), but obtained less draws to decision in the DXM group than all other groups. Whereas following HAL intervention the number of draws increased significantly compared to any other experimental group (all p≤0.048). Analyzing the probability estimates condition we quantified changes of probability estimates on an individual subject level whenever there was a change of bead color in a sequence (so called disconfirmatory evidence score, DES). In case of easy and difficult trial types we observed significantly higher DES scores in participants with SZ compared to HC (p≤0.003) and again obtained no differences between SZ and DXM (p=0.037).

**Discussion:**

Our findings are supportive for a hypothesized relationship between neurotransmitter state alterations of glutamate and dopamine in patients with schizophrenia and the delusional ideation. Future analysis will focus on developing a computational behavioral model of cognitive processing of the Beadstask, implementing our neurobiological findings in order to further disentangle the neurobiological underpinnings of delusional ideation in patients with schizophrenia.

